# Thin Solid Electrolyte Separators for Solid-State Lithium–Sulfur Batteries

**DOI:** 10.1021/acs.nanolett.2c04216

**Published:** 2022-12-16

**Authors:** Soochan Kim, Yvonne A. Chart, Sudarshan Narayanan, Mauro Pasta

**Affiliations:** † Department of Materials, University of Oxford, Oxford OX1 3PH, United Kingdom; ‡ The Faraday Institution, Quad One, Harwell Science and Innovation Campus, Didcot OX11 0RA, United Kingdom

**Keywords:** solid-state Li−S battery, thin solid electrolyte separator, Li_6_PS_5_Cl, XNBR

## Abstract

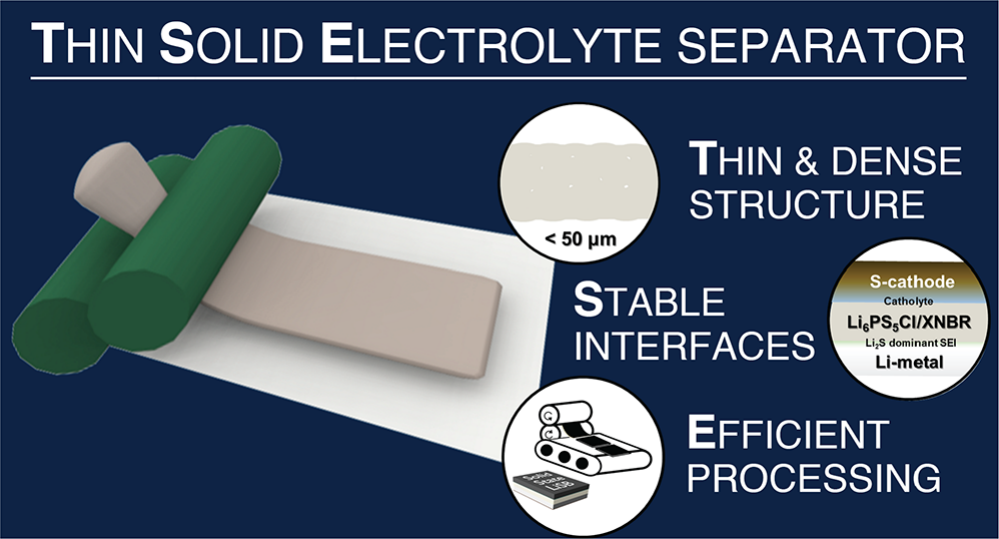

The lithium–sulfur battery is one of the most promising “beyond Li-ion” battery chemistries owing to its superior gravimetric energy density and low cost. Nonetheless, its commercialization has been hindered by its low cycle life due to the polysulfide shuttle and nonuniform Li-metal plating and stripping. Thin and dense solid electrolyte separators could address these issues without compromising on energy density. Here, we introduce a novel argyrodite (Li_6_PS_5_Cl)–carboxylated nitrile butadiene rubber (XNBR) composite thin solid electrolyte separator (TSE) (<50 μm) processed by a scalable calendering technique and compatible with Li-metal. When integrated in a full cell with a commercial tape-cast sulfur cathode (3.54 mg_*S*_ cm^–2^) in the presence of an in situ polymerized lithium bis(fluorosulfonyl)imide-polydioxolane catholyte and a 100 μm Li-metal foil anode, we demonstrate stable cycling for 50 cycles under realistic operating conditions (stack pressure of <1 MPa and 30 °C).

The future of electric transportation relies on novel battery chemistries with higher energy density and lower cost than state-of-the-art Li-ion batteries.^1,2^ Lithium–sulfur batteries (LiSBs) are one of the most promising candidates due to their high theoretical capacity (1675 mAh g^–1^) and the abundance of sulfur in the Earth’s crust.^2−4^ Despite decades of research and development, the widespread application of LiSBs remains hindered by their rapid capacity fade caused by the polysulfide shuttle and poor Li-metal plating and stripping efficiency.^5,6^

Replacing the liquid electrolyte (LE) with a solid electrolyte (SE) is the most promising avenue to address these issues.^2,5,6^ Various materials have been investigated for use as SEs, and they all have unique pros and cons.^2^ Among these, polymers are easy to process, provide good interfacial contact with the active materials, and can suppress the polysulfide shuttle.^5^ Unfortunately, their limited ionic conductivity, narrow electrochemical stability window, and inability to prevent Li-filament growth owing to insufficient mechanical strength vastly limit their utility.^2^ On the other hand, inorganic ceramics possess the electrochemical and mechanical properties necessary to hinder both polysulfides and Li-filament growth.^2,5,7^ Sulfide SEs are particularly promising because of their compatibility with sulfur cathodes and superior Li-ion conductivity, which is comparable to LEs at room temperature, with the added advantage of being easily processable.^2,8^ From a mechanical properties standpoint, while their soft nature facilitates their densification, their brittleness hinders their fabrication in large and thin form factors.^8^ Therefore, composite sulfide SEs with an elastic polymer are key to achieving thin solid electrolyte (TSE) separators below 50 μm capable of addressing the aforementioned problems while preserving the superior energy density of the Li–S chemistry.^2,5,9^

Herein, we propose a TSE separator consisting of Li_6_PS_5_Cl (LPSCl) and a carboxylated nitrile-butadiene rubber (XNBR), manufactured by a scalable calendering process. LPSCl is one of the most promising sulfide SEs because of its reasonable interfacial compatibility with Li-metal anodes, its earth-abundant precursors, and its high ionic conductivity.^10,11^ XNBR has great potential for use as a binder for LPSCl composites due to its elastic and adhesive properties from the polar −CN and −COOH functional groups in its structure and compatibility with nonpolar solvents.^12^ Forming LPSCl composites with conventional polymer binders containing polar functional groups (e.g., polyvinylidene fluoride, polyacrylic acid) has been difficult due to the reactivity of LPSCl with the polar solvents required to dissolve these polymers.^13^ Thus, polymers which are soluble in less polar or nonpolar solvents were introduced as binder candidates, such as a nitrile-butadiene rubber and acrylate-types (details in Table S1).^13^ LPSCl–XNBR composites allow for the formation of a flexible, easily processable TSE separator with the ionic conductivity and Li-metal compatibility benefits of LPSCl.

Historically, solid-state LiSBs have shown poor electrochemical performance due to poor contact at the electrode–electrolyte interfaces and the electrically and ionically insulating nature of sulfur. These challenges can be mitigated by applying high stack pressures, using elevated operating temperatures, or decreasing the sulfur content in the cathode, leading to unrealistic operating conditions.^2,10,14,15^ We overcome these issues by incorporating a lithium bis(fluorosulfonyl)imide (LiFSI)-polydioxolane (PDOL) electrolyte integrated into the cathode (catholyte), prepared by in situ polymerization of 1,3-dioxolane within the sulfur cathode. The compliant and ionically conductive catholyte helps maintain physical contact and ionic pathways at the cathode–SE interfaces during cycling, thus making it possible to implement commercial tape-cast sulfur cathode with realistic sulfur loading. Under practical operating conditions (<1 MPa stack pressure and 30 °C), symmetric Li–Li cells cycled stably for over 500 h at 0.1 mA cm^–2^ (0.05 mAh cm^–2^), and Li–S full cells assembled with a catholyte-containing commercial cathode (S-loading, 3.54 mg cm^–2^; S-content, 70 wt % in cathode) showed stable cycling for over 50 cycles with specific capacity of 410 mAh g^–1^.

Figure 1a shows a schematic representation of the TSE fabrication process. LPSCl, XNBR (3–10 wt %), and toluene were mixed using a mortar and pestle to form a rubbery composite that could be easily calendered into thin films (Figure 1b). LPSCl has mechanical and electrochemical properties which are sufficient to mitigate Li-filament growth, possessing a relatively high room-temperature ionic conductivity of ∼1 mS cm^–1^.^2,16,17^ The addition of XNBR further improves the mechanical properties of LPSCl for scalable processing while being soluble in toluene, a nonpolar solvent that does not react with LPSCl. Toluene facilitates dispersion of the active material and polymer to form a uniform composite, removing the need for high-energy mixing and also acting as a lubricant to prevent adhesion of the composite to the roller. Meanwhile, its low boiling point facilitates its removal and prevents it from affecting the properties of the final composite (only <1 wt % remains postcalendaring, Figure S1). Moreover, in nonpolar solvents, the polar functional groups (−CN and −COOH) of XNBR are available to interact with the LPSCl surface forming intermolecular bonds that significantly increase the effectiveness of XNBR as a binder.^18−20^

**Figure 1:**
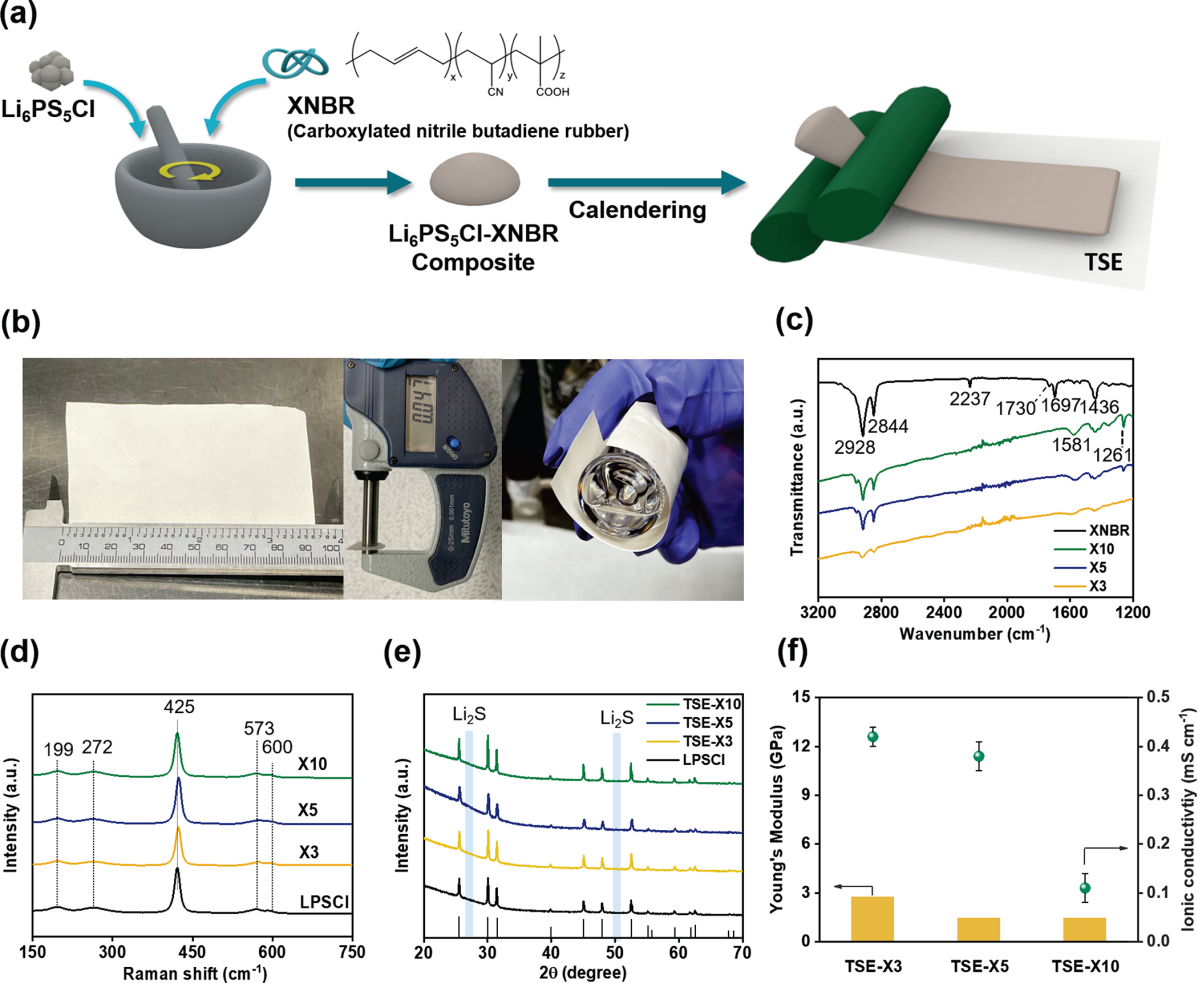
(a) Schematic of the TSE separator fabrication process with (b) pictures of the TSE separator showing a 9 × 6 cm^2^ area (left) with a thickness of 47 μm (center) and demonstrating its flexibility (right). Results of material characterization of prepared TSEs with 3, 5, and 10 wt % XNBR binder (TSE-X3, TSE-X5, TSE-X10, respectively) using (c) FT-IR spectroscopy, (d) Raman spectroscopy, and (e) XRD. (f) Results of testing the Young’s modulus by compression and ionic conductivity by EIS.

To determine a suitable binder content, we investigated TSE composites containing 3, 5, and 10 wt % XNBR binder (TSE-X3, -X5, and -X10, respectively). It was found that binder contents below 3 wt % were insufficient to collate the SE particles, resulting in flaky composites. Meanwhile, composites containing XNBR in excess of 10 wt % were too sticky to process. To analyze the composites resulting from this manufacturing process, several analytical techniques were used. Fourier transform infrared spectrometry (FT-IR) was used to investigate the effects of the binder on intermolecular bonding, as can be seen in Figure 1c. The addition of the binder results in a shift toward lower wavenumbers in the FT-IR spectra for the peaks at 1730 and 1697 cm^–1^, which are attributed to the carboxylic groups in XNBR (carbonyl stretching of mono and hydrogen-bonded carboxylic acid, respectively). The shift in the broad peaks near 1585 cm^–1^ demonstrates the formation of carboxylates (−COO^–^) due to the reaction between the electron-rich functional groups (−COOH) by electron-accepting sites such as P^5+^ and Li^+^.^18,19,21^ The shift in corresponding FT-IR peaks toward lower wavenumbers supports this interpretation and implies the formation of intermolecular bonds between LPSCl and XNBR.

In Figure 1d,e, Raman and XRD analyses were conducted to confirm the chemical and structural stability of the LPSCl in the TSEs. The Raman spectra exhibited peaks which can all be attributed to vibrational modes of the PS_4_^3–^ within LPSCl, as detailed in Table S2. From the XRD pattern of the SEs, prominent diffraction peaks (detailed in Supporting Information) are consistent with the pattern for pure LPSCl without any evidence of decomposition to Li_2_S.^20,22^ These results confirm that the LPSCl remains chemically stable during preparation of the TSEs. To find the optimum binder content, the mechanical and electrochemical properties of the TSEs were investigated as shown in Figure 1f. Increasing the binder content improves the flexibility (decrease in Young’s modulus) of the TSE, but as the binder is not ionically conductive, it also decreases its ionic conductivity (detailed in Table S3). Among the prepared TSEs, X5 was found to be optimal in terms of both ionic conductivity and Young’s modulus values. Whereas TSE-X3 exhibited a brittle nature with a high Young’s modulus (∼3 GPa), thereby making it vulnerable to mechanical shock, TSE-X10 showed poor ionic conductivity (∼0.1 mS cm^–1^). Therefore, TSE-X5 was used in subsequent investigations.

Sulfide SEs are known to undergo decomposition on contact with Li-metal, forming a Li-ion conductive interface consisting of Li_2_S, LiCl, and Li_*x*_P.^13,23^ This heterogeneous interface affects the Li plating and stripping behavior, potentially leading to lower interfacial ionic conductivity, nonuniform deposition, accelerated Li-filament growth, and degradation during battery cycling.^16^ Therefore, the interfacial stability between Li-metal and the TSE was evaluated using X-ray photoelectron spectroscopy (XPS), as can be seen in Figure 2. The result was then compared with those for a binder-free, pellet type-LPSCl SE prepared by cold pressing, as shown in Figure S2. XPS analysis was carried out with in situ deposition of Li-metal on the SE by an Ar^+^ ion beam to investigate the chemical evolution at the interface, as shown schematically in Figure 2a. The XPS spectra obtained from the TSE (Figure 2b) surface showed a gradual shift in the Li 1s spectra toward lower binding energies implying a reaction with the SE surface to form a solid electrolyte interphase (SEI).^16^ Further deposition leads to the appearance of a feature characteristic of metallic Li (Li^0^) species observed at a binding energy near 52.5 eV. Additionally, a doublet feature characteristic of Li_2_S (highlighted in orange) was noticed in the S 2p spectra. The formation of Li_2_S is consistent with the reported components of the SEI between Li-metal and LPSCl.^24,25^ Meanwhile, in the P 2p spectra, the formation of Li_*x*_P is difficult to observe, unlike in the SE pellet (Figure S2). These results demonstrate that the TSE forms a stable SEI in which Li_2_S was the dominant component in contact with Li-metal and was observably different from that formed with the SE pellet. Therefore, the XNBR also affects the interfacial chemistry and helps to improve its stability.

**Figure 2:**
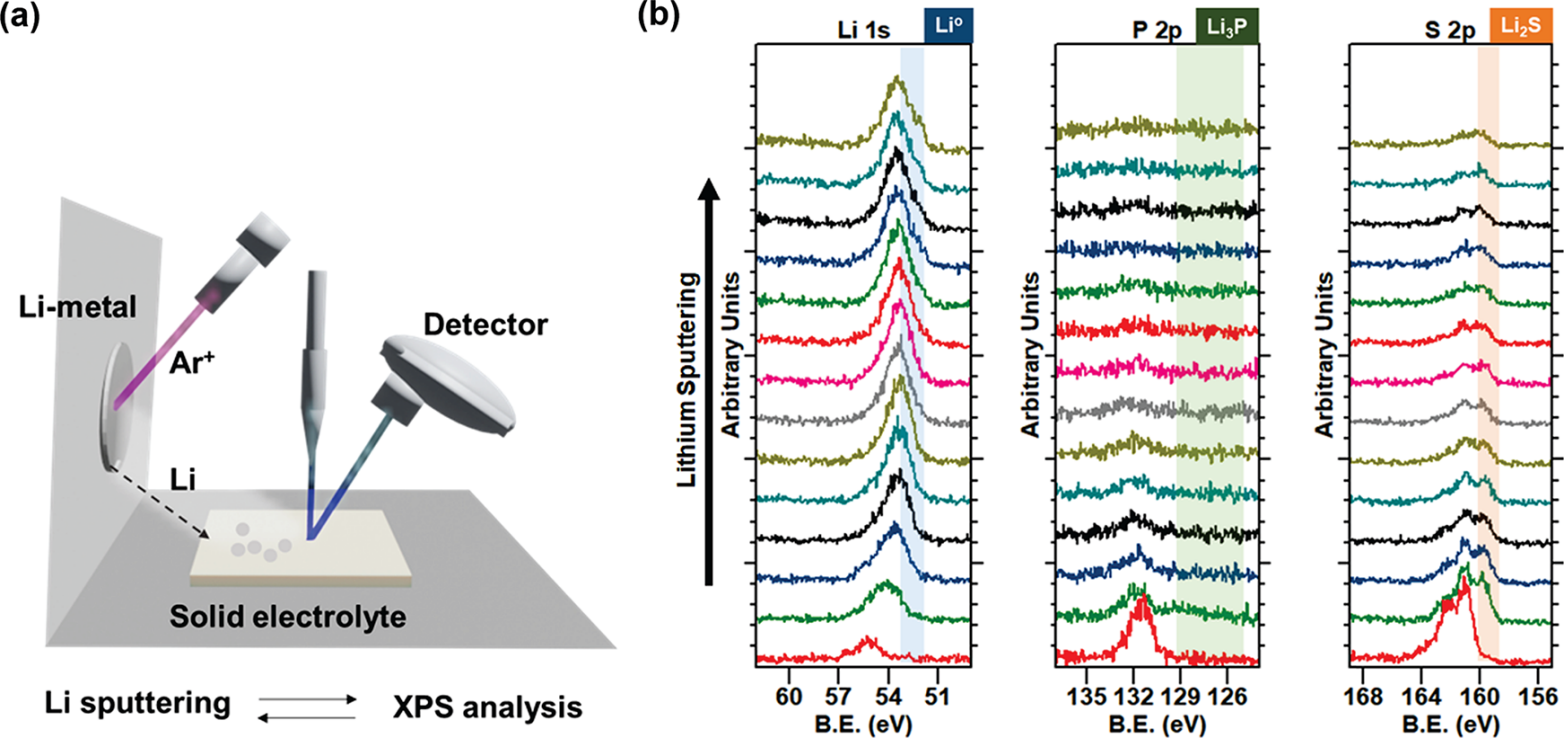
(a) Schematic of XPS analysis with in situ Li sputtering and (b) XPS spectra from the TSE with continual in situ Li-metal deposition.

To determine the limits of practical use for this SE system with a Li-metal anode, linear sweep voltammetry (LSV), electrochemical impedance spectroscopy (EIS), and critical current density (CCD) tests were run to investigate its electrochemical limitations. Figure 3a shows the electrochemical stability of the TSE separator measured via linear sweep voltammetry (LSV) at 0.1 mV s^–1^. To test the cell in a configuration similar to practical conditions, the LSV was conducted in a carbon-coated Al/TSE/Li-metal cell. At around 2.5 V, a small current increase of 200 nA cm^–2^ was observed, which can be ascribed to the oxidation of LPSCl to form an insulating interphase composed of Li_2_S, S, and P_2_S_*x*_ at the surface of the TSE in contact with the carbon-coated Al.^26,27^ After that, the cell was confirmed to be stable up to 5 V without any significant increases in current, due to the stability of formed Li_2_S.^28^ Moreover, the electronic conductivity of the TSE was evaluated by a chronoamperometry (at 0.2, 0.25, and 0.5 V) and measured as an average of 7.32 × 10^–11^ S cm^–1^, as shown in Figure S3a. This value was lower than the reported electronic conductivity of an LPSCl pellet (∼10^–10^ S cm^–1^) due to the use of the nonconductive polymer binder.^29,30^

**Figure 3:**
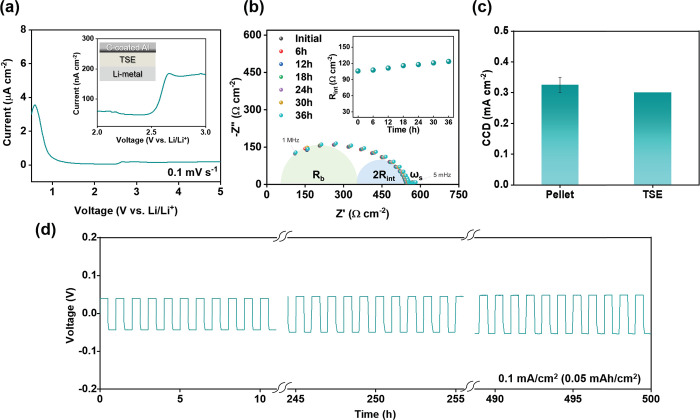
(a) LSV curves of carbon-coated Al/TSE/Li-metal cells from open-circuit voltage to 5.0 V, (b) Nyquist plots of Li/TSE/Li cells over time, (c) CCD of the pellet SE and TSE, and (d) cycling of a symmetric Li–Li cell with the TSE at 0.1 mA cm^–2^ for 500 h.

Symmetric Li–Li coin cells were assembled and evaluated under practical conditions (at 30 °C, <1 MPa inherent to the coin cell) to test the EIS and CCD with a SE pellet, and TSE. Figures 3b and S3b display the impedance spectra of the TSE and the SE pellet, respectively, assembled into symmetric Li/SE/Li cells. Using the equivalent circuit model in Figure S3c, the Nyquist plots were fitted to two RC circuits representing the two interfacial resistances (2 × *R*_int_) between the Li-metal anodes and SE and the bulk resistance of the SE (*R*_b_), and the Warburg impedance (ω_s_).^31−34^ Even after maintaining contact between Li-metal and the TSE for over 36 h, changes in *R*_int_ were insignificant, indicating a stable interface between the SE and Li (Figure 3b). Meanwhile, in the case of the SE pellet, *R*_int_ and *R*_b_ continually increased over the same time period, demonstrating the high reactivity between Li-metal and LPSCl (Figure S3b). These results are consistent with the formation of a stable SEI layer between TSE and Li-metal which is indicated in Figure 2b. Based on the stable electrochemical characteristics of the TSE with Li-metal, Li plating and stripping behavior were investigated with increasing current density from 0.01 to 0.4 mA cm^–2^ for each electrolyte (detailed in Figure S4 and Table S4). As shown in Figure 3c, on average the symmetrical Li–Li cell with a ∼ 600 μm thick SE pellet developed a short-circuit after applying 0.325 mA cm^–2^. Meanwhile, the <50 μm thick TSE (1/12 as thick as the pellet) demonstrated a similar CCD, 0.3 mA cm^–2^. This performance could be attributed to the dense microstructure of the TSE, with its low porosity (average 4.47%, detailed in Table S5).

In addition, to evaluate the stability of the TSE for use in long-term cycling, a symmetric Li–Li cell was assembled and cycled at 0.1 mA cm^–2^ (0.05 mAh cm^–2^) for 500 h. The overall trend and magnified voltage profiles are shown in Figures 3d and S5. After cycling, the overpotential and interfacial resistance were largely unchanged (Figure S5c), and the interface between TSE and Li-metal maintained good contact during repeated Li-plating and stripping, with no evidence of short circuit. Thus, this TSE is practical to manufacture commercially, shows good stability against Li-metal, and can withstand long-term battery cycling without failure.

Despite the intrinsic advantages of the TSE in terms of manufacturability and interfacial stability, direct integration into a practical Li–S cell has its own challenges. Whereas most reports on solid-state LiSBs using sulfide-based chemistries employ a system comprising pelletized layers (cathode composite/thick SE/anode) operating at high stack pressures (∼100 MPa), very few studies are able to demonstrate functional cells constructed using scalable approaches (e.g., tape-cast film-type cathode/SE/metal anode) that can be cycled under practically relevant conditions, i.e., stack pressures of <1 MPa, room temperature, and in the absence of any liquid electrolyte.^2,10^ In particular, solid-state battery architectures struggle with weak interfacial contact between the cathode and electrolyte, leading to poor electrochemical performance. While application of high stack pressure offers a means to mitigate these effects by maintaining and enhancing ionic and electronic conductivity across the interfaces,^35^ its implementation outside of laboratory setups is impractical.^10^ Moreover, the difficulty in fabricating composite cathodes microstructures with optimized ionic pathways often requires the use of large fractions of the SE material within the cathode mixture, thereby severely limiting the achievable energy density from cycling (detailed in Table S6).

To overcome these issues in a practical cell configuration, we introduce an integrated solid-state battery design with a commercial tape-cast sulfur cathode (BE-70E, NEI Corp.) containing a LiFSI-PDOL polymeric catholyte (details in Figure S6), as shown in Figure 4a. This was achieved by infiltrating 2 M LiFSI dissolved in 1,3-dioxolane (DOL) into the sulfur cathode upon complete assembly of the cell. The solution then undergoes in situ polymerization and forms PDOL, with the LiFSI acting as the initiator (Figure 4b).^36^ The catholyte provides ionic pathways within the cathode and TSE while also enhancing contact between these layers through improved wetting which can enable operation of the assembled cell under practically relevant stack pressures (<1 MPa) and at room temperature, which is a step toward an ideal solid-state battery system (Figure 4c).

**Figure 4:**
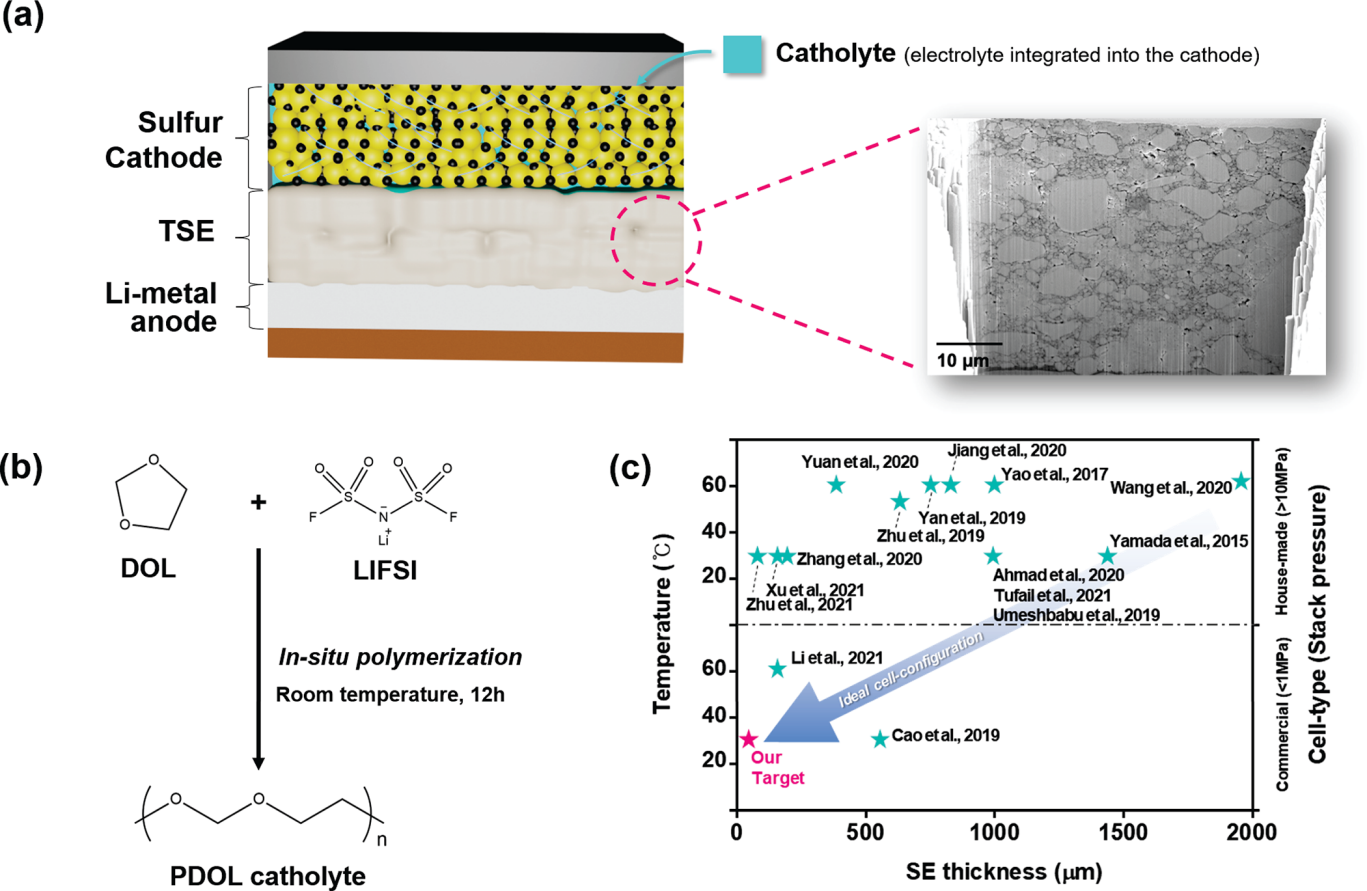
(a) Schematic of a solid-state Li–S battery system presented here with a FIB-SEM image of the TSE, (b) a diagram of the in situ polymerization process, and (c) recently reported cell-operating conditions of solid-state LiSBs with sulfide-based SEs (detailed in Table S6).

Based on this solid-state LiSB architecture, the TSEs were then assembled into full cells with the commercial S-cathode, a Li-metal anode (100 μm thick) and tested under practical conditions (<1 MPa stack pressure, at 30 °C). Figure 5a shows a comparison between the initial discharge profiles of LiSBs with a LE and TSE. While the Li–S cell with a LE showed two plateaus (the upper plateau represents the conversion from S_8_ to Li_2_S_4_, the lower from Li_2_S_4_ to Li_2_S), the solid-state Li–S cell with a TSE only produced one discharge plateau at ∼2.15 V, which indicates a direct reaction from S_8_ to Li_2_S (S + 2Li^+^ + 2e^–^ → Li_2_S), as is seen in conventional solid-state LiSBs with pellet SEs.^2,22,37−39^ Moreover, further investigations of the conversion processes were conducted by EIS in Figure 5b (detailed in Figure S7 with the explanations).

**Figure 5:**
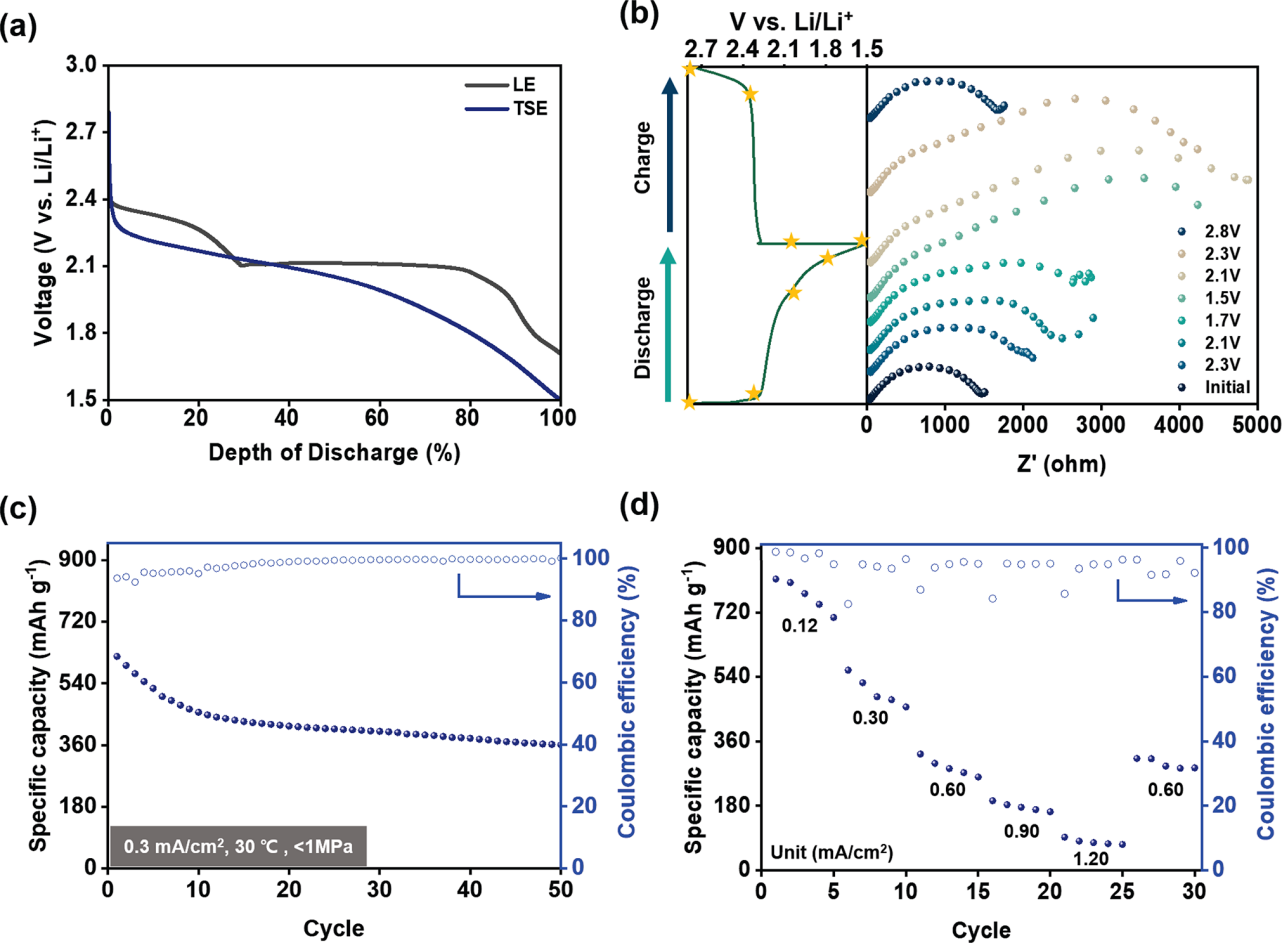
(a) Initial galvanostatic discharge curves of LiSBs with a LE (1 M lithium bis(trifluoromethanesulfonyl)imide with 0.8 M LiNO_3_ in a 1/1 (v/v) solution of 1,3-dioxolane and 1,2-dimethoxyethane) and TSE, (b) EIS characterization of solid-state LiSB with a TSE during its first cycle, where the left shows the points selected for EIS tests in the voltage profile and the right shows the EIS spectra at these points. (c) Battery cycling of a solid-state Li–S coin cell using a TSE at 0.30 mA cm^–2^ and (d) battery cycling at different current densities (0.12, 0.30, 0.60, 0.90, 1.20, and 0.60 mA cm^–2^).

Finally, the TSE was cycled in the solid-state LiSB coin cells at 0.3 mA cm^–2^, as shown in Figure 5c. The coin cell presented stable cycling and a high Coulombic efficiency (∼99%). Moreover, after 50 cycles, the solid-state LiSB showed a discharge capacity of 410 mAh g^–1^, which is comparable to Li–S cells using a LE (433 mAh g^–1^) or PDOL (183 mAh g^–1^), as shown in Figures S7b and S8. In Figure 5d, the prepared solid-state Li–S cell presented stable battery cycling at the different current densities from 0.12 to 1.20 mA cm^–2^. The cell delivered 722, 484, 314, 176, 90, and 310 mAh g^–1^ (at 0.12, 0.30, 0.60, 0.90, 1.20, and 0.60 mA cm^–2^, respectively) and showed excellent reversibility, even after the current density returned to 0.60 mA cm^–2^ (98.7% retention). This also indicates the formation of stable interfaces between the SE and cathode, without significant effects from the polysulfide shuttle to Li-metal or Li-filament growth. However, none of the assembled batteries achieved capacities near the theoretical value, including with the LE, as shown in Figure S7b. The early rapid loss of capacity was caused by the preformation of polysulfides during in situ polymerization as the commercial cathode used does not encapsulate the sulfur or include any special treatments which could prevent polysulfide leaching.^2,40^ These polysulfides initially act as active material but then diffuse into the catholyte where they are not electronically connected to the electrode.^2,41^ Therefore, an optimized cathode design is needed that can not only address these issues but also achieve the practical goals necessary for large-scale use of LiSBs: energy densities of >500 Wh kg^–1^ and high-rate capabilities. This could involve a dual-conductive surface coating, the use of redox mediators, 3D-electrode architecture, or advanced polymeric catholyte.^2−4,42−45^ Once this has been achieved, the full potential of this TSE separator can be realized, leading to a practical solid-state LiSB.

In summary, we have demonstrated a thin and scalable solid electrolyte separator and integrated battery system for operation under practical conditions. The thin solid electrolytes were prepared by calendering LPSCl–XNBR composites to a thickness of <50 μm and presented good compatibility with a Li-metal anode through the formation of stable interfaces. Within a solid-state LiSB, the use of an in situ polymerized catholyte in the tape-cast commercial sulfur cathode led to the manufacture of a scalable solid-state battery while maintaining a high sulfur content and improved physical/ionic contacts at the cathode–solid electrolyte interfaces. The prepared solid-state LiSBs exhibited stable cycling over 50 cycles under practical operating conditions. Further development of specialized cathodes to work with this system could enable realization of even higher capacities. This work demonstrates a major step toward commercial solid-state LiSB systems with a scalable manufacturing method and improved interface architecture.

## Supporting Information Available

Experimental details, photos of Li_6_PS_5_Cl–XNBR composites and PDOL, XPS spectra and Nyquist plots of a SE pellet, chronoamperometry profiles used to measure the electronic conductivity of the TSE, detailed symmetrical Li–Li cycling profiles, full profile of symmetrical Li–Li cycling, EIS characterization of the first cycle of a Li–S cell using a LE, battery cycling data of a Li–S cell using a LE and a PDOL, peak assignments of Li_6_PS_5_Cl in Raman spectroscopy, ionic conductivity and porosity of SEs, and summary of recently reported solid-state LiSBs which use a sulfide SE (PDF)
