# Supplementary material for: Thin Solid Electrolyte Separators for Solid-State Lithium–Sulfur Batteries
Source: Nano Lett. 2022 Dec 16;22(24):10176–83. doi: 10.1021/acs.nanolett.2c04216 (PMC9801416; doi:10.1021/acs.nanolett.2c04216)
Supplement: Supplementary file 1 — nl2c04216_si_001.pdf [file nl2c04216_si_001.pdf]

Supporting Information
Thin Solid Electrolyte Separators for Solid-State Lithium-Sulfur Batteries

Soochan Kim,[†,‡] Yvonne A. Chart,[†,‡] Sudarshan Narayanan,[†,‡] and Mauro Pasta*[,†,‡]

†*Department of Materials, University of Oxford, OX1 3PH, United Kingdom*

‡*The Faraday Institution Quad One, Harwell Science and Innovation Campus, Didcot OX11 0RA, United Kingdom*

E-mail: mauro.pasta@materials.ox.ac.uk

1. Experimental

Thin solid electrolyte (TSE) separator preparation

Li_6PS_5Cl (LPSCl) solid electrolyte (SE) powder (99.9 %, ~1 μm, Ampcera Inc., density=1.64 g/cm^3) and a carboxylated nitrile butadiene rubber (XNBR, Krynac X750, Arlanxeo, density=0.98 g/cm^3) binder (3 – 10 wt. %) were combined in anhydrous toluene (99.8 %, Sigma Aldrich, 110.6 °C of boiling point) and mixed using a vortex mixer or agate mortar and pestle. On mixing, a composite was formed which was then placed between Si-coated polyethylene terephthalate films (PET, thickness= 50 μm) and formed into a thin film *via* repeated calendaring. The thickness of the prepared TSE film was controlled by the calendaring machine settings (MTI corp., MSK-2150). After drying under vacuum at room temperature overnight, the film was cold-pressed with stainless steel plungers under a uni-

axial pressure of 200 MPa for 3 min. All processes were carried out in an Ar-filled glove box (MBRAUN, MB 200B, H_2O<0.1 ppm, O_2 <0.1 ppm).

Composite cathode preparation

A 2M solution of lithium bis(fluorosulfonyl)imide (LiFSI, 99.0 %, Fluorochem Ltd., dried under vacuum at 70 °C for 12 h) in 1,3-dioxolane (DOL, anhydrous 99.8 %, Sigma Aldrich, dried with molecular sieves for 24h) was infiltrated into a commercial, tape-cast sulfur cathode (BE-70E, NEI corp., 3.54 mg cm^{-2} of sulfur, 70 wt. % sulfur, 20 wt.% carbon black and 10 wt.% poly(vinylidene fluoride) binder). 10 μL of the solution per mg of sulfur in the cathode. The LiFSI acts as initiator for the polymerization reaction of DOL to form a poly-DOL solid polymeric catholyte. After infiltration, the assembled cells were stored at room temperature for 6h. All processing steps were conducted in an Ar-filled glove box.

Comparison electrolyte preparation

SE pellets were formed by pressing the same Li_6PS_5Cl powder as used in the TSE at 370 MPa for 3 min in a polyether ether ketone (PEEK) mould between stainless steel plungers. Liquid Li-S cells were assembled using 1M lithium bis(trifluoromethanesulfonyl) imide (LiTFSI) with 0.8M $LiNO_3$ in a 1/1 (v/v) solution of 1,3-dioxolane and 1,2-dimethoxyethane (Electrolyte/sulfur=15 μL/mg of S, DOL and DME dried using molecular sieves for 24 h, LiTFSI dried at 110 °C for 24 h, $LiNO_3$ dried at 120 °C for 24 h, all Sigma Aldrich) held within a polyolefin separator (Celgard 2500). The Li-S cells using a PDOL electrolyte consisted of 150 μL of a 2M LiFSI-DOL solution like that used for the composite cathode above, but infiltrated into a glass fiber separator (GF/F, Whatman).

Physical characterization

All characterization was conducted without exposure to air. To confirm the stability of the TSE, Fourier-transform infrared spectroscopy (FT-IR, NICOLET iS50, Thermo Fisher Scientific) was carried out in the 3200-1200 cm^{-1} wavenumber range using attenuated total reflection (ATR) mode. Each spectrum was measured by averaging 24 scans at a resolution of 2 cm^{-1}. X-ray powder diffraction (XRD, MiniFlex 600, Rigaku) was carried out under inert (nitrogen) gas with Cu Kα radiation. Patterns were collected from 20 to 70 ° 2θ at a scan rate of 1 ° min^{-1}.From the XRD pattern of the SEs, prominent diffraction peaks at 25.5°, 30.0°, 31.4°, 45.0°, 47.9°, and 52.4° were indexed to the (220), (311), (222), (422), (511), and (440) LPSCl planes, respectively. Raman measurements of the LPSCl powder and TSE separators were collected using an inVia Raman confocal microscope coupled with an inverted Leica microscope (Renishaw). A 633 nm laser was used as the excitation source (power <300 μW), focused onto the sample using a × 50 objective (Olympus). The samples were covered by glass and a polyimide film to avoid exposure to air. The collected Raman spectra were baseline corrected and peak fitted using a combined Lorentzian and Gaussian function. The spectral measurement time was 2 s with 24 accumulations to ensure a good signal to noise ratio to resolve peaks.

For XPS analysis, the SEs (pellet and separator) and Li metal were transferred to the XPS chamber using vacuum transfer vessels to eliminate air exposure. XPS spectra were collected using a Phi XPS VersaProbe III with an Al Kα X-ray source. For XPS measurements during in-situ Li deposition, sputtering of the Li-metal foil (Sigma Aldrich, thickness = 380 μm) was performed using an acceleration voltage of 2 kV and an Ar^+ beam current of 1 μA. The angle between the sputter gun and sample surface was 33°. The estimated sputtering depth rate was 0.7 Å min^{-1}, hence the thickness of Li after deposition was approximately 14 nm.

Plasma focused ion beam scanning electron microscopy (PFIB-SEM) sectioning was used to characterize the cross-section morphology of the electrode. PFIB sectioning was performed using a Thermo Fisher Helios G4-CXe Plasma-FIB. The samples were transferred to the

PFIB using an airless transfer vessel (Gatan), then milled and polished using a constant voltage of 30 kV and a current down to 15 nA.

Mechanical tests of TSE separators were performed in compression mode using a testing machine (Deben microtest, MT5000 with Dual Leadscrew Tensile Tester H-550 Controller, DEBEN UK Limited.) at 30 °C in an Ar-filled glove box. The samples tested had a 5 mm diameter and were placed on a stainless steel plunger within a PEEK sample holder. The test time was set to 0.5s, with the cross-head motor speed programmed to a displacement rate of 0.2 mm min^{-1}. The results presented show the average values of three samples.

Electrochemical characterization

To measure the ionic conductivity of the SE, electrochemical impedance spectroscopy (EIS) was carried out using a VMP-3 potentiostat (Biologic, France) with a voltage amplitude of 10 mV in the frequency range of 1 MHz to 0.01 Hz with the cell-configuration (carbon coated Al foil/SE/carbon coated Al foil). The ionic conductivity of the pellet SE (circular disk, 10mm diameter) was measured with a 9 MPa stack pressure using an in-house battery cell design[1] as well as under a stack pressure of < 1 MPa using a coin cell (CR2032, Hoshen Co., Ltd, Japan), both at 30 °C.

The electronic conductivity of the TSE was measured by the direct current (DC) polarization[2,3] with a cell-configuration of Ni/TSE/Ni symmetrical cells. Three voltages (0.2, 0.25, and 0.5 V) were applied to the symmetrical cells for 1 h, and the steady-state current (the change width of current was less than 0.5 nA) was recorded. From the recorded current, the resistance was calculated from the voltage-current curves, and the electronic conductivity was determined by considering the thickness and area of the TSE.

For the symmetric Li-Li cell cycling, two 100 μm thick Li-metal anodes with a diameter of 12.7 mm (100 μm thick, Sigma Aldrich) which were polished and calendared before use to remove any surface layers were attached to each side of the pellet SE or TSE separator.

Before cell assembly, 5 MPa of pressure was applied to this configuration for 1 min to affix the Li-metal to the SE and then assembled into a coin cell. The electrochemical resistance of the symmetrical cells was conducted by the EIS with a voltage amplitude of 10 mV in the frequency range of 1 MHz to 0.005 Hz. The symmetric cells were cycled under $<$1 MPa at 30 °C with various current densities as mentioned in the results section.

Li-S full cells were assembled in coin cells using commercial cathodes (BE-70E, NEI corp.) along with a Li-metal anodes as described above. Solid Li-S cell cycling was carried out between 1.5 and 2.8 V (vs. Li/Li^{+})at a constant 0.05 C rate, using a BCS-800 battery cycler (Biologic, France). Liquid Li-S cell cycling was carried out between 1.7 and 2.8 V (vs. Li^{+}/Li) at a constant, 0.05 C rate. All the electrochemical characterization was carried out at 30 °C.

2. Figures and tables

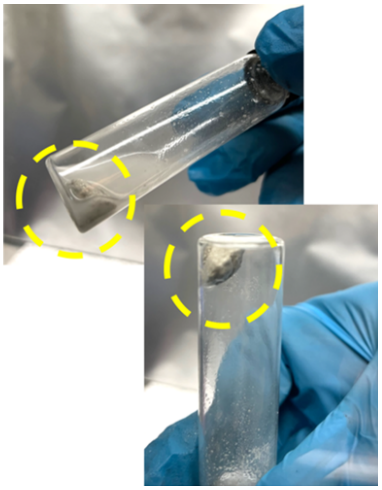

Figure S1: Images of the prepared LPSCl-XNBR composites in non-polar media (toluene)

During the mixing process, the LPSCl powder and XNBR solution easily form a LPSCl-XNBR composite in non-polar media.

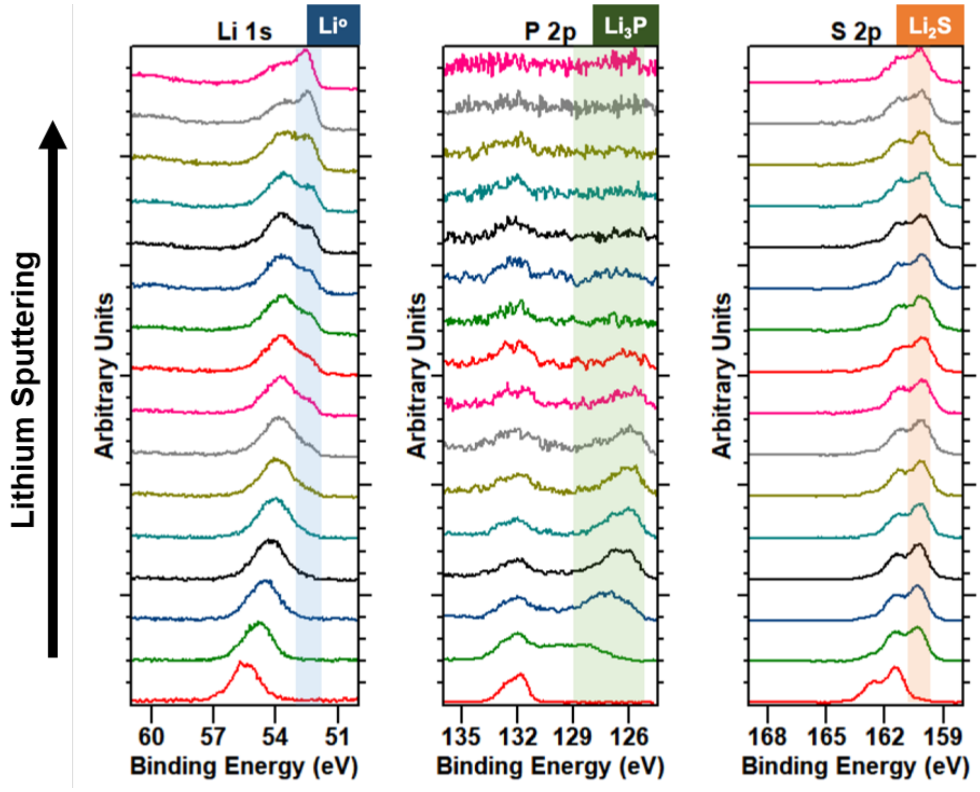

Figure S2: XPS spectra of a SE pellet with in-situ Li sputtering over time

In the case of a SE pellet, Li_xP and Li_2S were formed on contact with Li-metal.

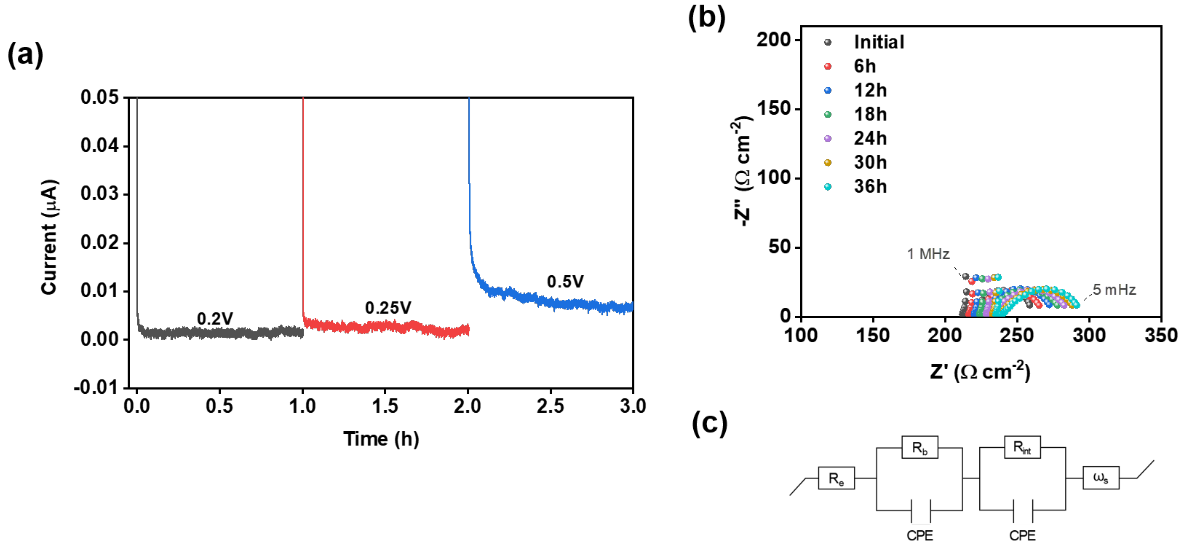

Figure S3: (a) Chronoamperometry profiles used to measure the electronic conductivity of the TSE, (b) Time evolution of Nyquist plots of Li/SE pellet/Li cell, and (c) Equivalent circuit model used to fit data in Figure 3b and Figure S3b.

An electronic conductivity of the TSE was measured the direct current (DC) polarization with a cell-configuration of Ni/TSE/Ni symmetrical cells.[2,3] Three voltages (0.2, 0.25, and 0.5 V) were applied to the symmetrical cells for 1 h, and the steady-state current (the change width of current was less than 0.5 nA) was recorded as 1.83×10^{-6}, 2.27×10^{-6}, and 5.28×10^{-6} mA, respectively. From the recorded currents, the electronic conductivity was calculated by the voltage-current curves and the thickness and area of the TSE.

An equivalent circuit model consisting of (R_e), (R_bCPE_b), ($R_{int}CPE_{int}$), and (ω_s) was constructed to fit the EIS data, where R_{int}, R_b, R_E, and ω_s are the resistance of the interface between the Li-metal and SE, the bulk resistance of the SE, athe solid-electrolyte resistance, and Warburg impedance, respectively. CPE denotes a constant phase element.[4,5]

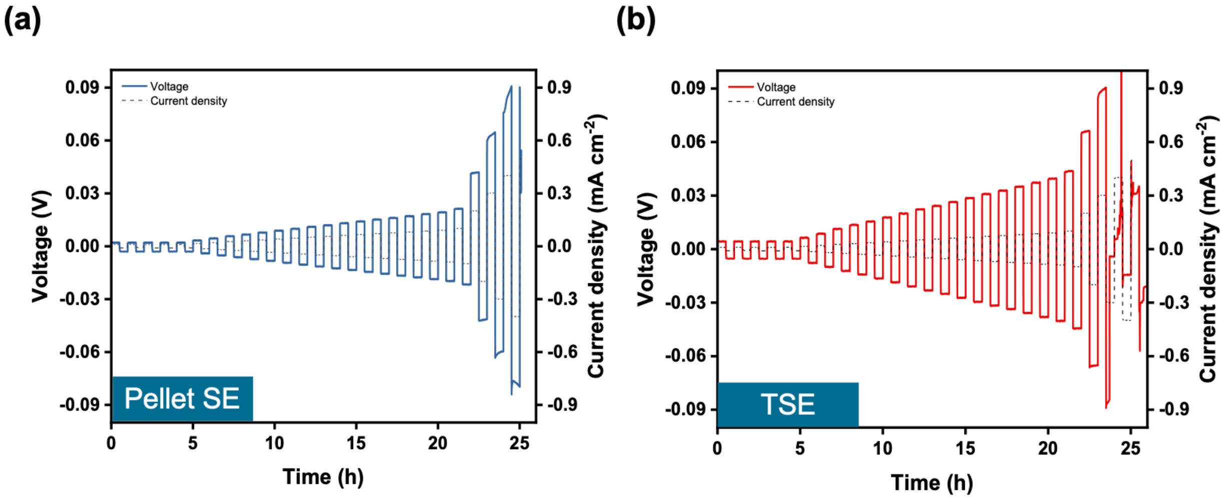

Figure S4: Detailed symmetrical Li-Li cycling profiles for (a) pellet SE and (b) TSE separator cells

To evaluate the critical current density of the prepared SEs, galvanostatic cycling of Li-Li cells was carried out at step-increased current densities at 30 °C. After activation cycles at 0.01 mA cm^{-2} for 4h, the current density was increased in 0.015 mA cm^{-2} steps from 0.01 to 0.1 mA cm^{-2}, after which the step size was decreased to 0.1 mA cm^{-2} up to 0.4 mA cm^{-2}. All cells eventually showed evidence of Li filament growth, as evidenced by voltage fluctuations or rapid voltage drops.

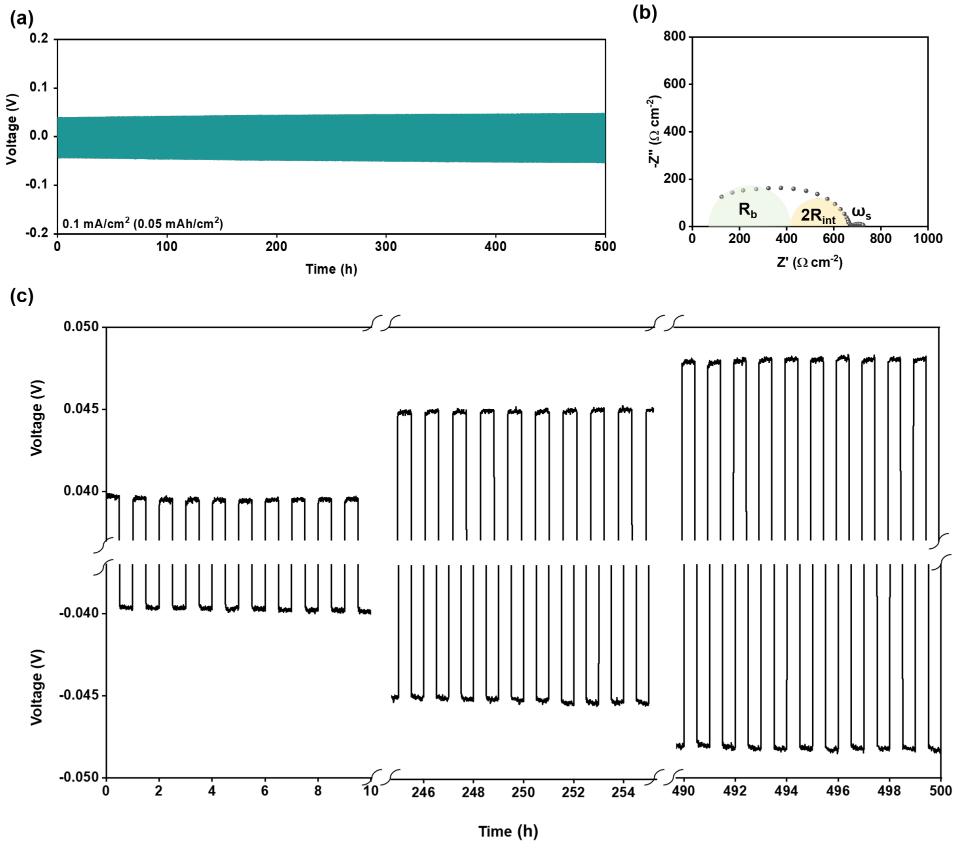

Figure S5: (a) Full profile of symmetrical Li-Li cycling, expanded from the information in Figure 3d, (b) Nyquist plot of a symmetrical Li-Li cell with a TSE separator after 500h of cycling, and (c) Top and bottom of the magnified voltage profiles extracted from Figure 3d

After 500 h of cycling at 0.05 mAh cm^{-2}, the overpotential of this cell increased from 40.1 mV to 47.8 mV, without any significant change in the shape of the profile. This shows relatively stable cycling.

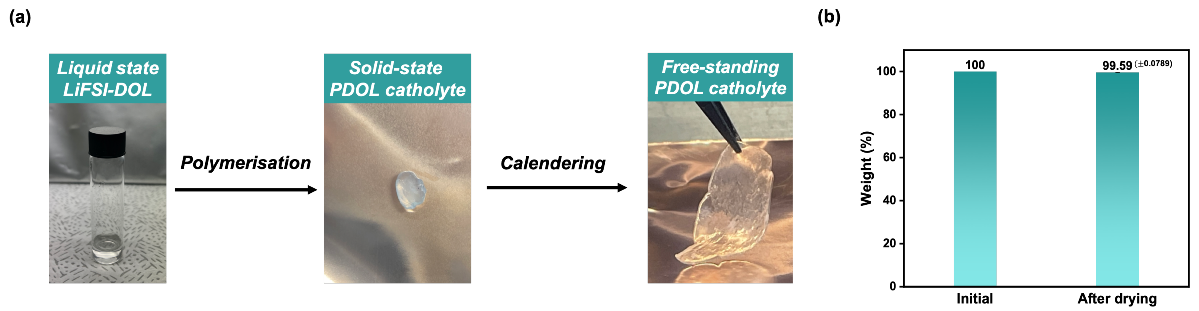

Figure S6: (a) Photos of the production of solid-state PDOL from a solution to a calendared, free-standing membrane, and (b) difference in weight of PDOL before and after drying

The choice of DOL as a soluble precursor along with the subsequent in-situ polymerization step here, is deliberate. Firstly, other non-polar solvents such as toluene, xylene, etc. easily dissolve sulfur-based materials, despite their apparent chemical compatibility with sulfide SEs.[6,7] This complicates fabrication of tape-cast type cathode composites with high sulfur contents. Secondly, DOL polymerizes into an ionically-conductive solid (ionic conductivity: ~ 0.308 mS cm^{-1} at 30 °C), maintaining the solid-state nature of the TSE at the end of the in-situ polymerization process. This is demonstrated by negligible change in weight of the catholyte when left to dry in vacuum at 80 °C over a 24h period. Moreover, the catholyte forms ionic pathways within the cathode and TSE, while also enhancing contact between these layers through improved wetting, as has been previously reported.[8,9] The infiltration of PDOL in this manner effectively enables operation of the assembled cell under practically relevant stack pressures (<1 MPa) and at room temperature, which is a step toward an ideal solid-state battery system

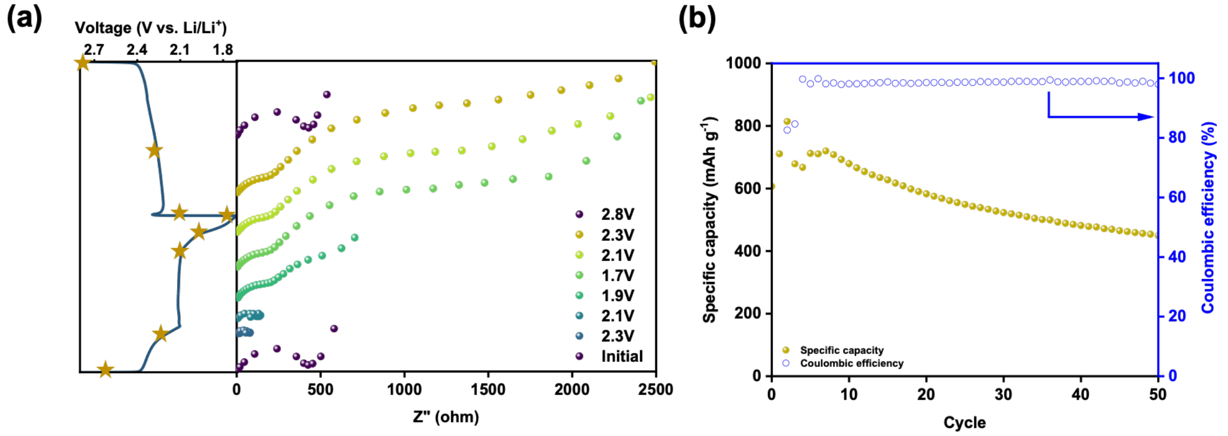

Figure S7: (a) EIS characterization of the first cycle of a Li-S cell using a liquid electrolyte; The selected points for the EIS tests are shown in the voltage profile on the left, with the respective EIS spectra on the right. (b) Longer term cycling of a Li-S full cell with a LE at 0.05 C (0.30 mA cm^{-2})

To further investigate the reaction processes occurring through discharging and charging, EIS spectra were collected at various points in the charge-discharge cycle for the cells with a TSE and LE as shown in Figures S7a, respectively. The direct solid-solid conversion reaction with the TSE caused the absolute change in the resistance to be much larger than with the LE. During discharge from the initial state to 1.5 V, the charge transfer resistance gradually increased due to direct conversion of solid S_8 to Li_2S, while the presence of a soluble liquid acting as an intermediary meant that the LE cell demonstrated lower intermediate resistances. On recharge, the impedance spectra reverted to a shape similar to that of the initial state, indicating the conversion of Li_2S back to S_8.[10] This shows that the TSE can reversibly undergo cycling without significant changes in resistance or trapping of S-species.

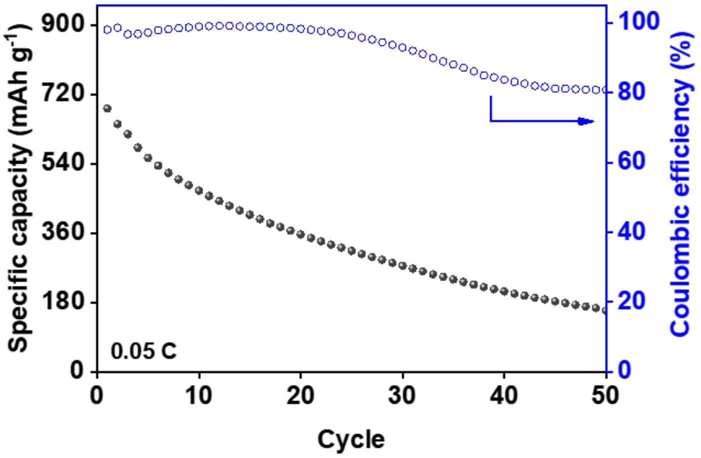

Figure S8: Battery cycling of a Li-S coin cell using a pure PDOL electrolyte at 0.05 C (0.30 mA cm^{-2})

Table S1: Reported polymeric binders for Li_6PS_5Cl solid electrolyte in the scalable process

**Polymer**	**Solvent**	**Thickness**	**Ionic conductivity**	**Ref.**
Poly (ethylene oxide)	Acetonitrile	65μm	0.2 mS cm^{-1}	[11]
Acrylate type polymer	Xylene/ISB	30μm	1.3 mS cm^{-1}	[12]
PTBA-b-BR	ISB	150μm	1.7 mS cm^{-1}	[13]
poly(butadiene methacrylate)	Xylene/ISB	40μm	0.1$\sim$0.8 mS cm^{-1}	[14]
Nitrile-butadiene rubber	Toluene/ISB	50μm	0.7$\sim$1.2 mS cm^{-1}	[15]
Ethyl cellulose	Toluene	47μm	1.6 mS cm^{-1}	[16]

*Isobutyl isobutyrate; ISB
*poly(tert-butyl acrylate)-b-poly(1,4-butadiene); PTBA-b-BR

Table S2: Peak assignments for the LPSCl in Raman spectroscopy

**Frequency (cm^{-1})**	**Assignment**
199	$PS_4{}^{3-}$
272	δ_{def} (S-P-S) in $PS_4{}^{3-}$
425	ν_s $PS_4{}^{3-}$ in $PS_4{}^{3-}$
573	$PS_4{}^{3-}$
600	$PS_4{}^{3-}$

The Raman spectra exhibited peaks at 199, 272, 425, 573, and 600 $^{-1}$ across all samples, which can all be attributed to vibrational modes of the PS_4^{3-} within LPSCl, as has been previously reported in literature.[17]

Table S3: Ionic conductivity of prepared SEs at 30 °C

**SE**	**Ionic conductivity (mS cm^{-1})**
**Under 9 MPa (stack pressure)**	
Pellet	0.79 (±0.04)
TSE-X3	0.42 (±0.02)
TSE-X5	0.38 (±0.03)
TSE-X10	0.11 (±0.03)

Table S4: Critical current density of SE in symmetrical Li-Li cell

**SE**	**Critical Current Density (mA cm^{-2})**
Pellet SE	0.325 ($\pm$0.025)
TSE	0.3 ($\pm$0)

Table S5: Porosity of SE

SE	Porosity
TSE before densification	13.15(±0.12)
TSE after densification	4.46 (±0.14)

The porosity of the SE was determined using equation (1). W_{TSE} and V_{TSE} are the weight and volume of the TSE separator, respectively. χ_a, and ρ_a are the weight fraction and density of component a in the composite.[18]

Equation (1):

$$\varepsilon(porosity, \%) = \{1 - \frac{W_{TSE}}{V_{TSE}} \times (\frac{\chi_{LPSCl}}{\rho_{LPSCl}} + \frac{\chi_{Polym.}}{\rho_{Polym.}}) \times 100\}$$

Table S6: Summary of recently reported solid-state Li-S batteries which use a sulfide SE

**Kinds of SE**	**SE thickness (μm)**	**Cathode (S-content %)**	**Anode**	**Stack Pressure**	**Temp. °C**	**Ref.**
Li_3PS_4	1412	S/SE/CF[a] (30)	Li	-	RT[b]	Yamada et al., 2015[19]
$Li_{10}GeP_2S_{12}$	1000	rGO[c]@S/SE/CB[d] (15)	Li	-	60	Yao et al., 2017[20]
$Li_{10}GeP_2S_{12}$+IL[e] (*Quasi solid-state*)	600	S/SE/CF/IL (40)	Li	Low (<1MPa)	RT	Cao et al., 2019[21]
$Li_{10}GeP_2S_{12}$+IL	-	S@C/SE/CB (20)	Li	-	RT	Umeshbabu et al., 2019[22]
Li_3PS_4 in Kevlar	100	Li_2S/LiI/CF/SE (36.9)	Li	-	RT	Xu et al., 2019[23]
$Li_7P_3S_{11}$	750	Li_2S@C/SE/CB (38)	Li-In	-	60	Yan et al., 2019[24]
Li_2S-P_2S_5/PEO[f] composite	120	S@CNT[g]/SE (24)	Li-In	High (100MPa)	RT	Zhang et al., 2020[25]
$Li_{10}GeP_2S_{12}$ or Li_6PS_5Cl	380	S@C/CB/SE (20)	Li-In	-	60	Yuan et al., 2020[26]
$Li_{3.25}Ge_{0.25}P_{0.75}S_4$/PEO composite	100	S@PAN[h]/CB (45) /PEO-LiTFSI	Li	Low (<1MPa)	60	Li et al., 2020[27]
Li_3PS_4	-	Li_2S/SE/CB (56.3)	Li-In	-	60	Jiang et al., 2020[28]
Li_3PS_4	800	Li_2S@SE@C (33)	Li	-	60	Jiang et al., 2020[29]
Li_3PS_4	-	Li_2S@SE/CB/CNT (50)	Li-In	-	60	Jiang et al., 2020[30]
$Li_{6.988}P_{2.994}Nb_{0.2}S_{10.934}O_{0.6}$	1000	Li_2S/SE/CF (30)	Li-In	-	RT	Ahmad et al., 2020[31]
Li_6PS_5Cl	1980	S/SE/CF (33)	Li	-	60	Wang et al., 2020[32]
Li_6PS_5Cl in cellulose	60	S/SE/CNT (25)	Li-In	-	RT	Zhu et al., 2021[33]
$Li_7P_{2.9}Ce_{0.2}S_{10}Cl_{0.3}$	-	Li_2S/SE/CB (30)	Li-In	-	RT	Zhou et al., 2021[34]
$Li_7P_{2.9}Sb_{0.1}S_{10.75}O_{0.25}$	-	S/SE/CB (18)	Li-In	-	60	Zhao et al., 2021[35]
$Li_7Sb_{0.05}P_{2.95}S_{10}I_{0.5}$	1000	Li_2S/SE/CF (30)	Li	-	60	Tufail et al., 2021[36]
Li_6PS_5Cl	-	S/SE/CB/PTFE[i] (25)	Li	-	60	Hu et al., 2022[37]
$Li_7P_3S_{11}$	645	S/SE/CB (30)	Li-In	High (110MPa)	50	Zhu et al., 2022[38]
Li_6PS_5Cl/XNBR composite	47	S/CB/PVDF binder (70)	Li	Low (<1MPa)	RT	Our work

[a]carbon fiber
[b]room temperature
[c]reduced graphene oxide
[d]carbon black
[e]ionic liquid
[f]polyethylene oxide
[g]carbon nanotube
[h]polyacylonitrile
[i]polytetrafluoroethylene

In Table S6, stack pressure is not specifically mentioned when house-made solid-state cell is used.

References

(1) Doerrer, C.; Capone, I.; Narayanan, S.; Liu, J.; Grovenor, C. R. M.; Pasta, M.; Grant, P. S. High Energy Density Single-Crystal NMC/Li6PS5Cl Cathodes for All-Solid-State Lithium-Metal Batteries. *ACS Applied Materials & Interfaces* **2021**, *13*, 37809–37815.

(2) Han, F.; Westover, A. S.; Yue, J.; Fan, X.; Wang, F.; Chi, M.; Leonard, D. N.; Dudney, N. J.; Wang, H.; Wang, C. High electronic conductivity as the origin of lithium dendrite formation within solid electrolytes. *Nature Energy* **2019**, *4*, 187–196.

(3) Lee, J. M.; Park, Y. S.; Moon, J.-W.; Hwang, H. Ionic and Electronic Conductivities of Lithium Argyrodite Li6PS5Cl Electrolytes Prepared via Wet Milling and Post-Annealing. *Frontiers in Chemistry* **2021**, *9*, 778057.

(4) Schlenker, R.; Stępień, D.; Koch, P.; Hupfer, T.; Indris, S.; Roling, B.; Miß, V.; Fuchs, A.; Wilhelmi, M.; Ehrenberg, H. Understanding the Lifetime of Battery Cells Based on Solid-State Li6PS5Cl Electrolyte Paired with Lithium Metal Electrode. *ACS Applied Materials & Interfaces* **2020**, *12*, 20012–20025.

(5) Wang, C.; Deng, T.; Fan, X.; Zheng, M.; Yu, R.; Lu, Q.; Duan, H.; Huang, H.; Wang, C.; Sun, X. Identifying soft breakdown in all-solid-state lithium battery. *Joule* **2022**, *6*, 1770–1781.

(6) Lee, J.; Lee, T.; Char, K.; Kim, K. J.; Choi, J. W. Issues and Advances in Scaling up Sulfide-Based All-Solid-State Batteries. *Accounts of Chemical Research* **2021**, *54*, 3390–3402.

(7) Ren, Y.; Shui, H.; Peng, C.; Liu, H.; Hu, Y. Solubility of elemental sulfur in pure organic solvents and organic solvent–ionic liquid mixtures from 293.15 to 353.15K. *Fluid Phase Equilibria* **2011**, *312*, 31–36.

(8) Zhao, Q.; Liu, X.; Stalin, S.; Khan, K.; Archer, L. A. Solid-state polymer electrolytes with in-built fast interfacial transport for secondary lithium batteries. *Nature Energy* **2019**, *4*, 365–373.

(9) Cheng, H.; Zhu, J.; Jin, H.; Gao, C.; Liu, H.; Cai, N.; Liu, Y.; Zhang, P.; Wang, M. In situ initiator-free gelation of highly concentrated lithium bis(fluorosulfonyl)imide-1,3-dioxolane solid polymer electrolyte for high performance lithium-metal batteries. *Materials Today Energy* **2021**, *20*, 100623.

(10) Wang, Y.; Liu, T.; Estevez, L.; Kumar, J. Kinetics of all-solid-state sulfur cathodes. *Energy Storage Materials* **2020**, *27*, 232–243.

(11) Luo, S.; Wang, Z.; Fan, A.; Liu, X.; Wang, H.; Ma, W.; Zhu, L.; Zhang, X. A high energy and power all-solid-state lithium battery enabled by modified sulfide electrolyte film. *Journal of Power Sources* **2021**, *485*, 229325.

(12) Lee, Y.-G. et al. High-energy long-cycling all-solid-state lithium metal batteries enabled by silver–carbon composite anodes. *Nature Energy* **2020**, *5*, 299–308.

(13) Lee, J.; Lee, K.; Lee, T.; Kim, H.; Kim, K.; Cho, W.; Coskun, A.; Char, K.; Choi, J. W. In Situ Deprotection of Polymeric Binders for Solution-Processible Sulfide-Based All-Solid-State Batteries. *Advanced Materials* **2020**, *32*, 2001702.

(14) Chen, Y.-T.; Duquesnoy, M.; Tan, D. H. S.; Doux, J.-M.; Yang, H.; Deysher, G.; Ridley, P.; Franco, A. A.; Meng, Y. S.; Chen, Z. Fabrication of High-Quality Thin Solid-State Electrolyte Films Assisted by Machine Learning. *ACS Energy Letters* **2021**, *6*, 1639–1648.

(15) Emley, B.; Liang, Y.; Chen, R.; Wu, C.; Pan, M.; Fan, Z.; Yao, Y. On the quality of tape-cast thin films of sulfide electrolytes for solid-state batteries. *Materials Today Physics* **2021**, *18*, 100397.

(16) Cao, D.; Li, Q.; Sun, X.; Wang, Y.; Zhao, X.; Cakmak, E.; Liang, W.; Anderson, A.; Ozcan, S.; Zhu, H. Amphipathic Binder Integrating Ultrathin and Highly Ion-Conductive Sulfide Membrane for Cell-Level High-Energy-Density All-Solid-State Batteries. *Advanced Materials* **2021**, *33*, 2105505.

(17) Zhou, Y.; Doerrer, C.; Kasemchainan, J.; Bruce, P. G.; Pasta, M.; Hardwick, L. J. Observation of Interfacial Degradation of Li6PS5Cl against Lithium Metal and LiCoO2 via In Situ Electrochemical Raman Microscopy. *Batteries & Supercaps* **2020**, *3*, 647–652.

(18) Sedlmeier, C.; Kutsch, T.; Schuster, R.; Hartmann, L.; Bublitz, R.; Tominac, M.; Bohn, M.; Gasteiger, H. A. From Powder to Sheets: A Comparative Electrolyte Study for Slurry-Based Processed Solid Electrolyte/Binder-Sheets as Separators in All-Solid-State Batteries. *Journal of The Electrochemical Society* **2022**, *169*, 070508.

(19) Yamada, T.; Ito, S.; Omoda, R.; Watanabe, T.; Aihara, Y.; Agostini, M.; Ulissi, U.; Hassoun, J.; Scrosati, B. All Solid-State Lithium–Sulfur Battery Using a Glass-Type P2S5–Li2S Electrolyte: Benefits on Anode Kinetics. *Journal of The Electrochemical Society* **2015**, *162*, A646–A651.

(20) Yao, X.; Huang, N.; Han, F.; Zhang, Q.; Wan, H.; Mwizerwa, J. P.; Wang, C.; Xu, X. High-Performance All-Solid-State Lithium–Sulfur Batteries Enabled by Amorphous Sulfur-Coated Reduced Graphene Oxide Cathodes. *Advanced Energy Materials* **2017**, *7*, 1602923.

(21) Cao, Y.; Zuo, P.; Lou, S.; Sun, Z.; Li, Q.; Huo, H.; Ma, Y.; Du, C.; Gao, Y.; Yin, G. A quasi-solid-state Li–S battery with high energy density, superior stability and safety. *Journal of Materials Chemistry A* **2019**, *7*, 6533–6542.

(22) Umeshbabu, E.; Zheng, B.; Zhu, J.; Wang, H.; Li, Y.; Yang, Y. Stable Cycling

Lithium–Sulfur Solid Batteries with Enhanced Li/Li10GeP2S12 Solid Electrolyte Interface Stability. *ACS Applied Materials & Interfaces* **2019**, *11*, 18436–18447.

(23) Xu, R.; Yue, J.; Liu, S.; Tu, J.; Han, F.; Liu, P.; Wang, C. Cathode-Supported All-Solid-State Lithium–Sulfur Batteries with High Cell-Level Energy Density. *ACS Energy Letters* **2019**, *4*, 1073–1079.

(24) Yan, H.; Wang, H.; Wang, D.; Li, X.; Gong, Z.; Yang, Y. In Situ Generated Li2S–C Nanocomposite for High-Capacity and Long-Life All-Solid-State Lithium Sulfur Batteries with Ultrahigh Areal Mass Loading. *Nano Letters* **2019**, *19*, 3280–3287.

(25) Zhang, Y.; Chen, R.; Wang, S.; Liu, T.; Xu, B.; Zhang, X.; Wang, X.; Shen, Y.; Lin, Y.-H.; Li, M.; Fan, L.-Z.; Li, L.; Nan, C.-W. Free-standing sulfide/polymer composite solid electrolyte membranes with high conductance for all-solid-state lithium batteries. *Energy Storage Materials* **2020**, *25*, 145–153.

(26) Yuan, H.; Nan, H.-X.; Zhao, C.-Z.; Zhu, G.-L.; Lu, Y.; Cheng, X.-B.; Liu, Q.-B.; He, C.-X.; Huang, J.-Q.; Zhang, Q. Slurry-Coated Sulfur/Sulfide Cathode with Li Metal Anode for All-Solid-State Lithium-Sulfur Pouch Cells. *Batteries & Supercaps* **2020**, *3*, 596–603.

(27) Li, M.; Frerichs, J. E.; Kolek, M.; Sun, W.; Zhou, D.; Huang, C. J.; Hwang, B. J.; Hansen, M. R.; Winter, M.; Bieker, P. Solid-State Lithium–Sulfur Battery Enabled by Thio-LiSICON/Polymer Composite Electrolyte and Sulfurized Polyacrylonitrile Cathode. *Advanced Functional Materials* **2020**, *30*, 1910123.

(28) Jiang, H.; Han, Y.; Wang, H.; Zhu, Y.; Guo, Q.; Jiang, H.; Zheng, C.; Xie, K. Facile synthesis of a mixed-conductive Li2S composites for all-solid-state lithium-sulfur batteries. *Ionics* **2020**, *26*, 4257–4265.

(29) Jiang, H.; Han, Y.; Wang, H.; Guo, Q.; Zhu, Y.; Xie, W.; Zheng, C.; Xie, K. In situ

generated Li2S-LPS composite for all-solid-state lithium-sulfur battery. *Ionics* **2020**, *26*, 2335–2342.

(30) Jiang, H.; Han, Y.; Wang, H.; Zhu, Y.; Guo, Q.; Jiang, H.; Zheng, C.; Xie, K. Li2S–Li3PS4 (LPS) Composite Synthesized by Liquid-Phase Shaking for All-Solid-State Lithium–Sulfur Batteries with High Performance. *Energy Technology* **2020**, *8*, 2000023.

(31) Ahmad, N.; Zhou, L.; Faheem, M.; Tufail, M. K.; Yang, L.; Chen, R.; Zhou, Y.; Yang, W. Enhanced Air Stability and High Li-Ion Conductivity of Li6.988P2.994Nb0.2S10.934O0.6 Glass–Ceramic Electrolyte for All-Solid-State Lithium–Sulfur Batteries. *ACS Applied Materials & Interfaces* **2020**, *12*, 21548–21558.

(32) Wang, Q.; Chen, Y.; Jin, J.; Wen, Z. A new high-capacity cathode for all-solid-state lithium sulfur battery. *Solid State Ionics* **2020**, *357*, 115500.

(33) Zhu, G.-L.; Zhao, C.-Z.; Peng, H.-J.; Yuan, H.; Hu, J.-K.; Nan, H.-X.; Lu, Y.; Liu, X.-Y.; Huang, J.-Q.; He, C.; Zhang, J.; Zhang, Q. A Self-Limited Free-Standing Sulfide Electrolyte Thin Film for All-Solid-State Lithium Metal Batteries. *Advanced Functional Materials* **2021**, *31*, 2101985.

(34) Zhou, L.; Tufail, M. K.; Ahmad, N.; Song, T.; Chen, R.; Yang, W. Strong Interfacial Adhesion between the Li2S Cathode and a Functional Li7P2.9Ce0.2S10.9Cl0.3 Solid-State Electrolyte Endowed Long-Term Cycle Stability to All-Solid-State Lithium–Sulfur Batteries. *ACS Applied Materials & Interfaces* **2021**, *13*, 28270–28280.

(35) Zhao, B.-S.; Wang, L.; Chen, P.; Liu, S.; Li, G.-R.; Xu, N.; Wu, M.-T.; Gao, X.-P. Congener Substitution Reinforced Li7P2.9Sb0.1S10.75O0.25 Glass-Ceramic Electrolytes for All-Solid-State Lithium–Sulfur Batteries. *ACS Applied Materials & Interfaces* **2021**, *13*, 34477–34485.

(36) Tufail, M. K.; Zhou, L.; Ahmad, N.; Chen, R.; Faheem, M.; Yang, L.; Yang, W. A novel air-stable Li7Sb0.05P2.95S10.5I0.5 superionic conductor glass-ceramics elec-

trolyte for all-solid-state lithium-sulfur batteries. *Chemical Engineering Journal* **2021**, *407*, 127149.

(37) Hu, J.-K.; Yuan, H.; Yang, S.-J.; Lu, Y.; Sun, S.; Liu, J.; Liao, Y.-L.; Li, S.; Zhao, C.-Z.; Huang, J.-Q. Dry electrode technology for scalable and flexible high-energy sulfur cathodes in all-solid-state lithium-sulfur batteries. *Journal of Energy Chemistry* **2022**, *71*, 612–618.

(38) Zhu, X.; Jiang, W.; Zhao, S.; Huang, R.; Ling, M.; Liang, C.; Wang, L. Exploring the concordant solid-state electrolytes for all-solid-state lithium-sulfur batteries. *Nano Energy* **2022**, *96*, 107093.
